# Digital chest X-ray through a mobile van: public private partnership to detect sputum negative pulmonary TB

**DOI:** 10.1186/s13104-017-2420-4

**Published:** 2017-02-14

**Authors:** Bornali Datta, Anupama Hazarika, Hemant Deepak Shewade, Kavita Ayyagari, Ajay M. V. Kumar

**Affiliations:** 1 Department of Respiratory Disease and Sleep Medicine, Medanta-The Medicity, Gurgaon, India; 2 International Union Against Tuberculosis and Lung Disease (The Union), South-East Asia Office, C-6 Qutub Institutional Area, New Delhi, 110016 India; 3 International Union Against Tuberculosis and Lung Disease (The Union), Paris, France

**Keywords:** Diagnostic algorithm, Public–private partnership, RNTCP, Chest radiography, CXR, Smear negative pulmonary TB, India

## Abstract

Nearly half of the smear negative pulmonary TB in National TB Programme remain undetected in Haryana (north Indian state), probably due to poor access to chest radiography. A corporate hospital stepped into fill this infrastructure gap in Rewari district of Haryana by sending a mobile van with digital x-ray facilities and paramedic staff. The staff of the public health facility coordinated with the eligible patients and ensured that they visited on the designated day. The District TB Officer interpreted the x-ray and made decisions about diagnosis and treatment. The support was provided between May and Dec 2014 in seven public health centres (primary/secondary level) of the district. A total of 355 patients were examined, of whom 122 (34.4%) were diagnosed as smear negative pulmonary TB and started on treatment according to programme guidelines. This public–private partnership needs to be scaled-up and better designed studies are required to assess community-level impact and cost-effectiveness.

## Background

Efforts in TB control in India in the past two decades have made remarkable progress in controlling TB and caring for patients with TB. Despite these gains India continues to have the highest burden of TB in the world with an estimated 2.8 million incident cases annually [1]. Haryana is a state in north India with a population of 25.3 million. Under revised national tuberculosis control programme (RNTCP), in Haryana, there are 21 reporting districts, 77 tuberculosis units at sub-district level, and 256 designated microscopy centres. Rewari is a reporting district in Haryana with a population of 0.93 million. The annual TB case notification rate (CNR) in this district is 125/100,000 people [2].

**Fig. 1:**
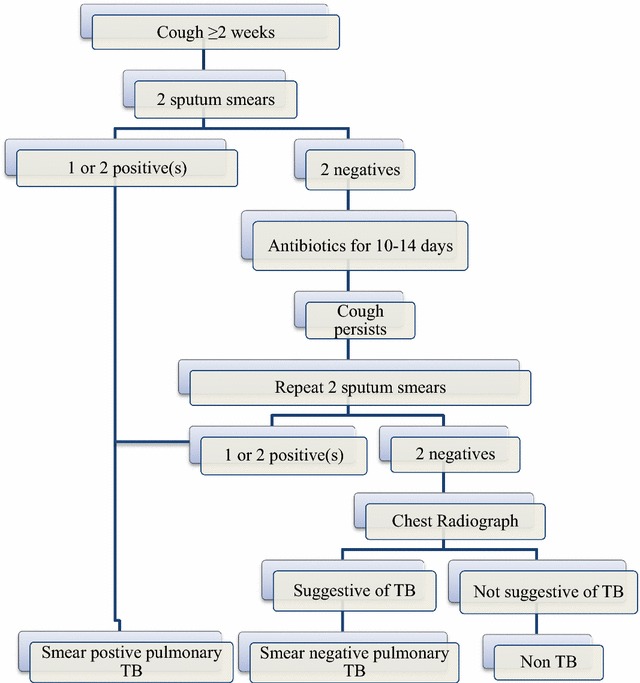
Revised national tuberculosis control programme diagnostic algorithm for pulmonary TB, India (2009)

According to RNTCP diagnostic algorithm (Fig. 1), sputum-negative chest symptomatic adults who do not improve with a 10–14 day course of antibiotics are eligible for a repeat sputum smear examination. Patients with second negative result are eligible for a chest radiograph (CXR). If the CXR is suggestive of TB, then a diagnosis of sputum-negative pulmonary TB is made [3].

Medanta-The Medicity is one of India’s largest multi-super specialty institution located in Gurgaon, a district in Haryana [4]. Medanta, a corporate hospital, collaborated with the reporting district of Rewari to support the implementation of RNTCP. Medanta has partnered with International Union Against Tuberculosis and Lung Disease (The Union) for “Call to Action for a TB Free India” in its effort to create and sustain high-level domestic commitment to end TB in India [5].

## Aspect of interest

Although the ratio of smear positive and smear negative TB cases was expected to be 1:1, the ratio in Haryana was 2:1 and remained skewed towards detection of smear positive cases [6]. In Rewari too, although the case notification of smear positive cases was 63/100,000, the smear negative case notification was 24/100,000 [2]. Thus, data suggested that more than half of the smear negative cases remained undetected or if they were detected, were not being notified to the Government notification system. The former could be due to limited access to chest radiography at peripheral health facility and there was anecdotal evidence that patients referred to the district level health facility were lost-to-follow-up.

To address this, Medanta-The Medicity implemented a pilot project in partnership with the District RNTCP team of Rewari, Haryana from May to December 2014. Under this public–private partnership, a mobile unit consisting of a mobile van and digital CXR machine was sent once a week to one Government health facility. The CXR machine was an Allenger’s 30 mA portable machine. Fuji digital reader was connected for digital review and printing of the CXR. The cost of the portable x-ray machine was ≈3000 USD. The cost of the digital reader was 9000 USD (however this was already there in the van for the existing mammogram). This van was staffed with a CXR technician, a radiographer and a driver. The team was led by a Senior Consultant of the Respiratory Medicine Department, Medanta–the Medicity. Seven Government health facilities [Primary Health Centre (PHC)/Community Health Centre (CHC)] namely CHC Khol, PHC Dharuhera, PHC Dahina, PHC Kasola, PHC Gudiyani, PHC Tankri, and PHC Jatusana were conveniently chosen and sequentially covered over the seven month period.

The medical officer of the health facility was informed in advance of the arrival of the mobile van. Adult patients (18 years and above) who according to the RNTCP diagnostic algorithm were eligible but not able to get a CXR were requested to assemble at the health facility on the designated date of visit of mobile unit. The Government health system staff ensured that the eligible patient made the visit. The chest x-ray (single view) interpretation was done by the District TB Officer who is a qualified chest physician and the lead of TB in the district. Those with findings consistent with active TB (apical infiltrates, cavity, miliary nodules, pleural effusion—in corroboration with appropriate clinical findings) were diagnosed as smear negative pulmonary TB and were initiated on treatment as per RNTCP guidelines.

Nineteen field visits were made by the mobile unit: six to PHC Dharuhera, four to CHC Khol, three to PHC Dahina, and two each to PHC Jatusana, PHC Gudiyani and PHC Tankri. A total of 355 patients were eligible for chest radiograph and were referred by the health centre to the mobile unit. Their mean (SD) age was 45 (17) years and 190 (54%) were males. The symptomatology was the following: cough for more than 14 days in 344, fever in 137 (39%), hemoptysis in 13 (4%), and weight loss in 99 (28%) cases. Based on digital radiograph, 122 (34.4%) were diagnosed as smear negative pulmonary TB. This intervention did not have an impact on the annual CNR of district Rewari (Table 1).

**Table 1:** TB case notification (total and smear negative pulmonary TB) in district Rewari, Haryana (India) in 2013 and 2014 [2, 6]

Case notification rate (CNR)^a^	Year	CNR	Percentage change from baseline
Total	2013	138	
	2014	125	
	Difference (2014–2013)	−13	−9
Smear negative pulmonary TB	2013	31	
	2014	24	
	Difference (2014–2013)	−7	−23

Digital X-ray through a mobile van/intervention was between May and Dec 2014

^a^CNR per lakh population

## Discussion

This pilot suggested that there was scope for provision of digital CXR through mobile vans as a part of public private partnership to detect smear negative pulmonary TB patients. The strength of this pilot is that in this public private partnership, the corporate private hospital had provided services to support the public health system to increase diagnosis of tuberculosis. The corporate hospital sent the mobile van with digital x-ray facilities along with paramedic staff. As there was active involvement of the district TB team (district TB Officer was involved in diagnosis) and Government health staff, there were no hurdles in initiating the patients with TB on treatment. These findings contrast with other documented public private partnerships in the past from India that focused only on training of private providers to refer or notify the cases to public health system [7]. The mobile van provided far greater outreach by bringing services to the community. This possibly addressed the challenges associated with accessing CXR which include long distances to reach district hospital and unavailability and/or non-functional CXR machines in the peripheral health centres.

While the intervention filled a gap, it did not impact CNR. This could be due to low coverage of the intervention—only 7 (39%) out of 18 public health facilities in the district were covered and implementation of intervention only for seven months of the year. On the other hand, over diagnosis of TB cannot be ruled out. A larger systematically implemented intervention might be expected to have a community-level impact. We did not collect patient-wise data systematically to comment on feasibility (proportion of chest symptomatic eligible for CXR evaluation and proportion among them receiving CXR through mobile unit) and effectiveness of the intervention. Future studies should be designed to make this possible.

Several studies in the past have demonstrated the inefficiencies in the existing diagnostic algorithm (Fig. 1). One issue is related to the complexity of the algorithm—with several steps and time required for each step—this often led to patient loss-to-follow-up [8]. Our intervention did not address the loss to follow-up that happens during the antibiotic trial. There is a need to simplify the algorithm and use better diagnostics. One possible way might be to provide CXR upfront in the diagnostic algorithm (immediately after initial smear negative result) and if suggestive of TB, be confirmed using a rapid test like Xpert MTB/RIF [9]. Also, this approach has high positive predictive value, low number to screen and is efficient but resource intensive [10]. The Union has recommended adding Xpert machines to the mobile van that could improve case detection rates in Haryana. This needs to be piloted in future and scaled-up if found effective to realize the vision of TB-free India.

## Conclusions

Despite the limitations of current pilot intervention, provision of digital radiograph through a mobile unit by Medanta resulted in the filling up of an infrastructure gap: however, it did not improve detection of smear negative pulmonary TB at the district level for various reasons. This public–private partnership needs to be scaled-up and better designed studies are required to assess community-level impact and cost-effectiveness. Medanta and The Union plan to do this in the coming year.

## Abbreviations

TB: tuberculosis
RNTCP: revised national tuberculosis control programme
CNR: chest notification rate
CXR: chest radiograph
PHC: primary health centre
CHC: community health centre
